# Dismantling the structures of inequality: why we need feminist leadership in the health sector

**DOI:** 10.1136/bmj-2023-078927

**Published:** 2024-07-17

**Authors:** Sarah Hawkes, Rama Baru

**Affiliations:** 1 Global Health 50/50, Wellington House, Cambridge, UK; 2 UK Institute for Global Health, University College London, London, UK; 3 Centre of Social Medicine and Community Health, Jawaharlal Nehru University, New Delhi, India

## Abstract

Approaches to increasing women’s representation in senior leadership need to go beyond individual empowerment and adopt principles of social justice, argue **Sarah Hawkes** and **Rama Baru**

Since the term was first coined in the 1970s, much has been written about the “glass ceiling” effect—the phenomenon whereby women and others who are subject to structural exclusion and marginalisation are present at all levels of the workforce except the top.^1^ The experience is so widespread that in the early 1990s the US government established a Glass Ceiling Commission, which ran for five years and sought to examine questions “relating to the advancement of women and minorities to management and decision-making positions.”^2^ The commission identified three types of barriers that were hindering equality at the highest levels of corporate America—societal barriers (eg, education levels, bias, and stereotyping), government barriers (lack of accountability systems), and organisational barriers (how businesses recruit, appoint, retain, and promote staff). More recently, the metaphor of the “glass cliff” has been used to describe the phenomenon in which women are disproportionately appointed to leadership positions that are “risky and precarious.”^3^

The findings from the American Federal Commission are extended in the articles in the BMJ collection on gender equality in the health workforce (www.bmj.com/collections/gender-equality-health-workforce). Thestudies examine factors across multiple levels, including individual, family, community, organisational, and legal, that have affected career progression in the health workforces of India and Kenya in particular, but with lessons drawn more widely.^4^ Articles in the collection also review accountability mechanisms that can be used to leverage change and hold both organisations and national governments to account for their commitments to equality and equity for women’s careers.^5^ The findings across these different cultures, contexts, and periods are consistent: inequitable career progression is driven by a mix of structural, social, organisational, and individual factors, and achieving career equality needs action across all levels. The research in India and Kenya further highlights the additional challenges specific to health sector workplaces: a female dominated workforce (except at the top) coupled with a history of occupational segregation that embeds and perpetuates gender inequalities—generally, nurses and midwives have been mainly female, and within health system hierarchies these have tended to be less powerful and less well paid occupations.^6^ Career inequalities in the health sector are further hampered by the pervasive fear or experience of harassment and violence from colleagues, patients, and visitors.^7^ ^8^ The persistence of inequality suggests that new approaches are needed to ensure better representation in healthcare leadership.

## Persistent inequality

Health sectors tend to exhibit what has been termed “inequality regimes”^9^—reflecting systematic and deeply embedded inequalities in workforces. Despite women comprising the majority of the healthcare workforce, they occupy only a quarter of senior roles.^10^ The lack of women’s progression to senior leadership is reflected in pay gap data. Analysis by the International Labour Organisation and World Health Organization finds a gender pay gap in the health and social care sector of 15-24%— bigger than the gaps in non-health sectors.^11^

Inequalities in health sectors go beyond gender: pay gap analysis in the UK NHS reports a mean gender pay gap of 11.4%, an ethnicity pay gap of 8.7%, and a disability pay gap of 6.9%.^12^ Inequalities start even before future staff are in the formal paid workforce. A 2011 study of academic attainment among students and staff trained in UK medical schools found significantly worse outcomes for non-white medics at both undergraduate and postgraduate levels.^13^

An extensive analysis of the public and private health sectors in India (where the private sector accounts for most service delivery and hence health worker employment) finds that people from middle and upper castes (social position determined by birth) are adequately or over-represented among health professionals while those from the most disadvantaged (scheduled) tribes and castes are significantly under-represented. The authors point out that private practices are mainly owned by upper and middle caste men, and the private sector health workforce has an over-representation of people from marginalised groups, who are often hired “to save labour costs with exploitative terms of work.”^14^

These national inequalities are replicated globally, including in global health organisations. Analysis of 146 major global health organisations found that only 17 (less than 1%) of more than 2000 board seats were occupied by women from low-income countries (compared with 882 (44%) seats occupied by Americans).^15^

Such inequalities in career progression persist despite universal commitments to workplace equal opportunities. Sustainable development goal (SDG) 5 on gender equality commits all signatory countries to “women’s full and effective participation and equal opportunities for leadership at all levels of decision-making in political, economic and public life.” This goal has seen the growth of activities across many of countries and sectors – including the health sector. For example, extensive work is underway in east Africa, India, and North America to “close the gender gap” in health sector leadership through WomenLift Health, an initiative funded by the Gates Foundation that supports mid-career women to develop their leadership potential.^16^ Numerous other gender equality initiatives exist, ranging from legally mandated non-discrimination laws,^5^ mandatory gender pay gap reporting (UK^17^), quota systems (eg, legally mandated quotas for board membership in Norway^18^), equality charter marks (eg, the Athena SWAN initiative^19^), and monitoring and accountability systems that hold organisations to account for their commitments and responsibilities to promote workplace equality.^20^

Approaches to increase women’s leadership often emphasise individual empowerment. Critics have highlighted that merely advocating for more women rising through the organisational ranks without addressing the unequal nature of the organisation or system itself risks reinforcing the idea that it is women not systems that need to be “fixed.”^21^ Drawing on these concerns, feminist academics and practitioners have advocated for a different approach to thinking about leadership, promoting a feminist leadership that emphasises principles of social justice and redistributive justice, including within organisations.^12^

Of course, who gets represented in leadership is an important first step towards social justice. However, this can quickly become subsumed into an emphasis on achieving parity targets rather than promoting social justice. We should also be seeking to achieve a more social justice oriented approach, promoting a style of leadership that enables a redistribution of power in an organisation, and recognises and respects all members of the workforce.^22^

While there are many styles and categories of leadership (box 1)—including some such as transformational or distributive leadership that “offer socially just possibilities,”^22^ we argue that feminist leadership is more likely to promote, support, and sustain the goal of social justice for all people working in the health sector. Calls for feminist approaches in health have been made before, including across the broad global health agenda,^23^ issues of global health security,^24^ and data for global health decision making.^25^ But what is feminist leadership and what difference can it make?

Box 1 **What is leadership?**

There is no universal definition of leadership, and merely rising to the top of an organisation may not always equate to holding power, particularly if an appointment is driven by token representation. Leadership is practised at every level of an organisation and is generally an expression of power—exerting influence, authority, and vision over others—but is also “giving voice to all people in an organisation.”^26^

While originally described in terms of the characteristics and skills of the leader, ideas about leadership changed in the mid-20th century with the focus shifting more towards the process of leadership, including the question of how a leader practises power.^27^

## How feminist leadership can make a difference

Feminist leadership is concerned with addressing and overturning the deep structures of inequalities that inhibit transformation towards more socially just organisations. It is not equivalent to simply having more women in leadership positions; nor is it “feminine” leadership styles—that is, compassion and support, in contrast to “masculine” traits of projecting power.^28^ Rather, feminist leadership is a politically aware process that speaks to questions of power—what it is, how it is distributed, how it can be shared, and how it is used for collective good. 

Feminist scholar Srilatha Batliwala identified four core components of feminist leadership: power, politics and purpose, principles and values, and practices.^29^ Feminist leadership seeks social justice and social transformation, including within organisations, and seeks to shine a light on and dismantle the multiple axes of exclusion (that is, going beyond gender to include ethnicity, class, disability, etc) and their intersection that maintain inequality within organisations.^9^

Feminist leadership could help achieve workplace focused social justice for all people working in health sectors—a concept that encompasses addressing and redressing inequalities, including those of power, privilege, and pay. Although the terminology of feminist leadership may be relatively recent, inclusive and transformational leadership is not. First Nations communities in Canada, for example, commonly practised matriarchal systems of leadership that had similar values to those seen in feminist leadership—a focus on systems and structures, redistributing power, and fostering collaboration.^30^

Measuring the effect of feminist leadership is fraught with challenges, and few studies have examined outcomes related to feminist leadership in the health sector. However, evidence from other social sectors shows the transformational possibilities inherent within feminist leadership.

In India, trade unions for Anganwadi and Accredited Social Health Activist (ASHA) community health workers have tackled entrenched inequalities to raise collective concerns of poor working conditions, terms of work, poverty wages, and sexual harassment. These two million workers are the bedrock of the Indian primary healthcare system, but their work has been recognised only as “voluntary” until recently, meaning they receive an honorarium and are not covered by the same formal legal employment rights as other health workers. Sustained agitation by these female workers has forced the formal health system to partially accede to their demands—including through directives from the Indian Supreme Court to improve working conditions and pay.^31^

In the 1990s, research identified feminist styles of leadership as integral to nursing that was participatory, problem solving, consensus driven, and person oriented and argued that effective [feminist] leadership was designed to be “empowering rather than threatening and coercive.”^32^ By the 2000s, interviews with American Black feminist leaders in community organisations working for people with HIV/AIDS found that leaders shared a “commitment to the value of creating positive and nurturing relationships,” motivating the powerless to feel more powerful, and a vision of shared responsibility towards a common goal. Interviewees promoted a view of leadership as inclusive and collective rather than the more traditional hierarchical ways of exerting power and exercising authority.^33^

Evidence from other sectors shows the potential benefits of applying feminist leadership In the education sector, for example, a feminist approach within leadership has been shown to produceseveral positive outcomes.^34^ It tackles intersectional inequalities, including ethnicity, among staff; promotes recognition and respect for difference both in the workforce and among school pupils, leading to an increased sense of belonging and social inclusion; and promotes the value of experiential knowledge and “student voice” in the classroom. There are important lessons here for the health sector where, for example, social exclusion has a measurable and important detrimental effect on health outcomes.^35^

## Putting feminist leadership into practice

Despite decades of evidence informed advocacy by a broad range of stakeholders including women’s movements, trade unions, and civil society organisations, the health sector remains inequitable for both patients and providers in most settings. In India, for example, robust evidence on the intersections between gender, caste, and class in the health services has not yet influenced any policy intervention that could further the ideas of feminist leadership to reduce inequities. This represents a missed opportunity but also raises the central question of how to practise feminist leadership in health sectors.

Despite the powerful arguments for leadership focused on social justice, studies of how to practise feminist leadership or its impact are rare, and most examples relate only to women as feminist leaders (that is, not questioning how people with other gender identities practise feminist leadership). This may reflect the challenges of practising social justice oriented leadership, identifying the metrics needed to evaluate processes and outcomes, and raising funding to conduct monitoring and evaluation. Some evidence can be gleaned from the work of feminist leaders reflecting on their own experience. For example, Fiona McKay, a university academic, writes of strategies that include practising “stealth feminism”—framing the feminist response as the norm rather than the exception—and forming strategic alliances with like-minded groups to achieve shared goals towards a more socially just workplace.^36^ Insightfully, McKay addresses the question of whether feminists can or should engage with systems and institutional structures that are fundamentally at odds with the principles of feminism, pragmatically concluding that non-engagement is unlikely to achieve progress towards the feminist goals we want to see.

The principles of feminist leadership can seem at odds with the dominant (neoliberal) ethos in many societies where health services are focused on metrics of efficiency and effectiveness, and where privatisation, profit, and competition are valued over notions of collective solidarity. In the face of such challenges feminist leadership is needed now more than ever. The metaphor of smashing the glass ceiling is an attractive rallying cry for reaching parity in leadership, but the ceiling rests on pillars and systems of inequality that require dismantling too. It is time for us not just to smash the ceiling but to rebuild the whole house according to a more feminist blueprint.

**Key messages**

Inequality in the health workforce is driven by a long history of unequal power relations based on characteristics such as gender, class, ethnicity, and disability
These inequalities are seen at the level leadership, with only certain kinds of people supported to rise to the top
Most efforts to increase representation (achieving parity, for example) focus on individuals rather than whether leaders will promote fairer workplaces for all
Embedding principles of feminist leadership in health sector organisations involves tackling the inequalities of power and privilege throughout the organisation
By doing so, feminist leadership can transform workplaces into more collaborative and socially just places which foster and support all staff on their career journeys

## Notes

Contributors and sources: This article accompanies a body of research work (under the same grant code) investigating the question of equality in career opportunities in the health workforces of India and Kenya. SH was the principal investigator for the larger project and is a specialist in gender and global public health. RB is a specialist in gender and health systems. The ideas for the article were jointly conceived and both authors contributed to, edited, and revised drafts of the paper. SH is the guarantor.

This article is part of the BMJ collection on gender equality in the health workforce ((www.bmj.com/collections/gender-equality-health-workforce), developed in partnership with Global Health 50/50, Africa Population and Health Research Centre, and International Center for Research on Women, and funded by the Bill and Melinda Gates Foundation. *The BMJ* commissioned, peer reviewed, edited, and made the decision to publish these articles. The lead editors were Seye Abimbola, Jocalyn Clark, and Emma Veitch for *BMJ Global Health* and *The BMJ*.
